# Preparation of hollow porous Cu_2_O microspheres and photocatalytic activity under visible light irradiation

**DOI:** 10.1186/1556-276X-7-347

**Published:** 2012-06-27

**Authors:** Yuan Yu, Liying Zhang, Jian Wang, Zhi Yang, Mingce Long, Nantao Hu, Yafei Zhang

**Affiliations:** 1 Key Laboratory for Thin Film and Microfabrication of the Ministry of Education, Research Institute of Micro/Nano Science and Technology, Shanghai Jiao Tong University, Shanghai 200240, China; 2 School of Environmental Science and Engineering, Shanghai Jiao Tong University, Shanghai 200240, China

**Keywords:** Cu_2_O, Hollow porous microspheres, Photocatalytic, Visible light

## Abstract

Cu_2_O p-type semiconductor hollow porous microspheres have been prepared by using a simple soft-template method at room temperature. The morphology of as-synthesized samples is hollow spherical structures with the diameter ranging from 200 to 500 nm, and the surfaces of the spheres are rough, porous and with lots of channels and folds. The photocatalytic activity of degradation of methyl orange (MO) under visible light irradiation was investigated by UV-visible spectroscopy. The results show that the hollow porous Cu_2_O particles were uniform in diameters and have an excellent ability in visible light-induced degradation of MO. Meanwhile, the growth mechanism of the prepared Cu_2_O was also analyzed. We find that sodium dodecyl sulfate acted the role of soft templates in the synthesis process. The hollow porous structure was not only sensitive to the soft template but also to the amount of reagents.

## Background

Much attention has been focused on fabricating high-efficiency photocatalytic materials, which is one of the most potential routes to mitigating environmental pollution [1]. Among them, metal oxide semiconductors, such as ZnO and TiO_2_, have attracted much attention owing to their high efficiency in the degradation of wide-ranged pollutants in which electron–hole pairs are generated under irradiation and degrade the pollutants absorbed on the surface of the photocatalytic materials [2-5]. Most of the metal oxide semiconductors have large band gaps, for example, 3.2 eV for ZnO [6] and 3.0 eV for TiO_2_[7], which are in the range of an UV spectrum. For this kind of materials, it is hard to generate electron–hole pairs under visible light irradiation because of the low photo energy, which therefore leads to lower photocatalytic efficiency and limits their large-scale applications. Therefore, in recent years, great efforts have been devoted to develop new photocatalytic materials with high efficiency under visible light irradiation.

As a typical p-type semiconducting material, cuprous oxide (Cu_2_O) possesses a stable, direct band gap of about 2.17 eV and a higher hall mobility up to 60 cm^2^/V·s [8], which has wide-scale applications in gas sensors, solar cells and lithium-ion batteries [7-9] owing to its unique optical and magnetic properties [9-15]. In 1998, Hara et al. [16] found that Cu_2_O can split water under visible light due to its low band gap. From then on, as a candidate of photocatalytic materials, Cu_2_O has attracted much research interests due to its important applications in degrading industrial dyeing wastewater, nitrogen-containing pesticides, etc. under visible light energy. Cu_2_O particles with different shapes, such as cube, octahedral, multipod, nanowire, hollow structure and porous spheres, have been synthesized [17-22]. It was found that the properties varied with the specific structure in different shapes [23]. Especially, hollow-structured particles with large specific areas have widespread potential applications in photocatalysts [24], drug delivery carriers, lightweight fillers and gas sensors [25,26]. Among lots of preparation approaches, the soft-template method is commonly used to synthesize Cu_2_O hollow-structured particles. So far, some polymer, such as EDTA-4Na [27], gelatin [28], PEG [29], PVP [30] and Oleic [31], were used as soft templates to fabricate hollow structures. Moreover, most reported photocatalytic studies related to Cu_2_O materials are mainly focused on UV-visible light irradiation [24,32,33]. In this paper, we used sodium dodecyl sulfate (SDS) as a soft template accompanied with gas bubble processes [34] to synthesize hollow porous Cu_2_O microspheres. The optical properties and photocatalytic activities under visible light irradiation were also investigated.

## Methods

All of the chemical reagents used in the experiment were of analytical reagent grade and were directly used without further purification. To prepare the hollow porous Cu_2_O particles, CuSO_4_ was used as the Cu^2+^ source with SDS as the soft template. In a typical procedure, 180 mg SDS was dissolved into 45 ml deionized water under magnetic stirring for more than 20 min to form a stable micellar solution. Then, 1 ml (0.1 g/ml) CuSO_4_ solution, 0.04 ml (13 M) ammonia and 0.15 ml (5 M) NaOH solution were added in sequence to the above solution every 20 min. The color of the solution then turned to light turbid blue from clarification. After fully stirred, 0.18 ml (50 wt.%) N_2_H_4_·H_2_O solution was added dropwise as the reducing agent. As the reaction proceeded with constant stirring, the solution produced a lot of bubbles. The whole experiment process was under the condition of 20 °C for 40 min. When the reaction finished, the final products were separated by centrifugation and cleaned several times by filtration with plenty of deionized water and ethanol. Finally, the products were dried at 50 °C for 6 h in a vacuum oven.

The crystal structures of the as-prepared samples were identified by X-ray powder diffraction (XRD) using an advanced X-ray diffractometer (D8 ADVANCE, Bruker, Bremen, Germany) with a Cu-Kα rotating anode point source operating at 40 kV and 40 mA. The morphology and size were investigated by field emission scanning electron microscopy (SEM; Zeiss Ultra 55, Carl Zeiss Microscopy GmbH, Hamburg, Germany) at an accelerating voltage of 5 kV. The inner microstructure of the as-synthesized samples was studied by transmission electron microscopy (TEM; JEM-2100, JEOL, Tokyo, Japan). The optical absorption properties of the as-prepared Cu_2_O microspheres were characterized by UV-visible absorption spectroscopy with a He-Cd laser line of 325 nm as an excitation source (Lab-RAM HR 800 UV, HORIBA Jobin Yvon, Kyoto, Japan). The photocatalytic activity was analyzed by using methyl orange (MO) as a model pollutant molecule. The photocatalytic absorbance measurements were performed on a UNIC7000 spectrophotometer (Unic Company, USA) at 464 nm.

## Results and discussion

**Figure 1:**
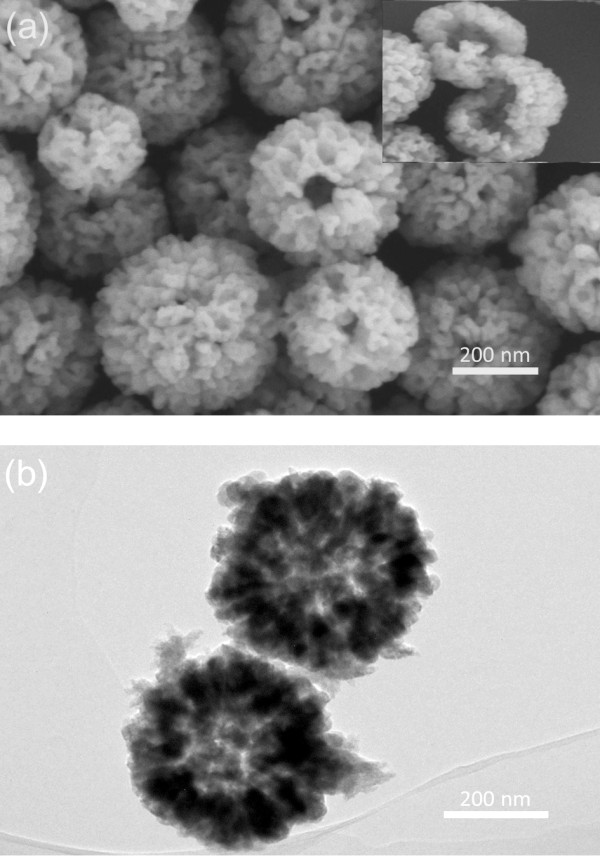
**SEM (a) and TEM (b) images of the as-prepared Cu**_**2**_**O hollow porous microspheres.** The inset in Figure 1a is an SEM image of several unclosed hollow spheres.

The SEM and TEM images of the as-prepared Cu_2_O hollow microspheres are shown in Figure 1a,b. The morphology of the sample has been identified as hollow spherical structures, and the surfaces of the spheres are rough, porous and with lots of channels and folds. Most of them are uniform, and the diameter ranges from 200 to 500 nm. The TEM result and SEM images of several unclosed particles (inset in Figure 1a) confirm the hollow structure further.

**Figure 2:**
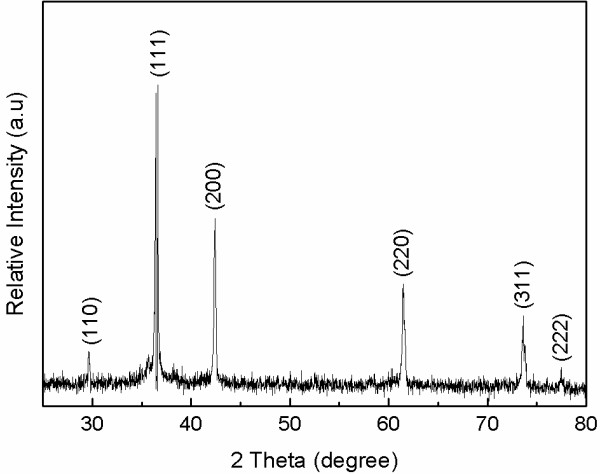
**XRD pattern of the Cu**_**2**_**O hollow porous microspheres.**

The typical XRD patterns of the as-synthesized hollow microspheres are shown in Figure 2. All the diffraction peaks of the samples are labeled and can be indexed very well according to the standard cubic phase Cu_2_O with a space group Pn3m (JCPDS file no. 05–0667). From the XRD patterns, no other characteristic diffraction peaks, such as CuO or Cu, can be detected, indicating that the pure Cu_2_O was obtained using this simple soft-template method at room temperature. The average crystalline grain size of the sample was calculated from the XRD patterns according to the Scherrer formula (*D*_hkl_ = *kλ* / *β*cos*θ*, where *D* is the average crystalline grain size, *k* is the Scherrer constant related to the shape and index (hkl) of the crystals, *λ* is the wavelength (0.154056 nm) of the X-ray, *θ* is the Blagg diffraction angle, and *β* is the full-width at half-maximum). The average crystalline grain size of the as-synthesized nanoparticle was estimated to be about 26 nm.

**Figure 3:**
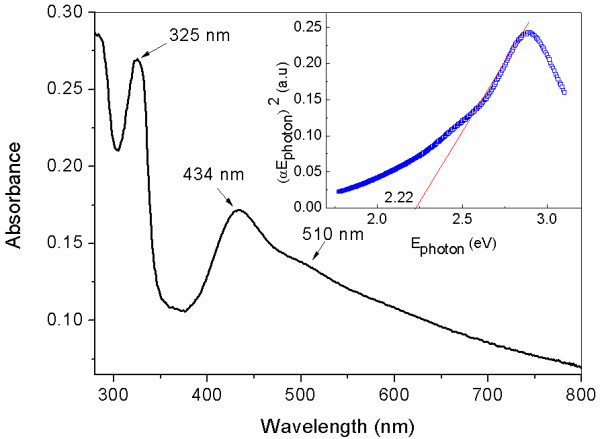
**UV–vis absorbance spectra of as-prepared hollow porous Cu**_**2**_**O microspheres.** The inset is the plot of (*αE*_photon_)^2^ versus *E*_photon_ to evaluate the band gap of Cu_2_O microspheres.

Optical absorption behavior is one of the very important fundamental properties in revealing the energy structures and applications in photocatalysis. Figure 3 shows the UV-visible absorbance spectra of the as-prepared hollow porous Cu_2_O spheres by ultrasonically dispersing in absolute ethanol. Two strong absorption peaks in the UV region are observed at wavelengths of about 434 and 325 nm for the as-synthesized samples. The broader one around 434 nm should attribute to the intrinsic band gap absorption, and the sharp one at 325 nm may result from the residual SDS absorption peak. The direct optical band gap energy (*E*_g_) of the Cu_2_O microspheres can be calculated from the 434-nm absorption peak. The inset in Figure 3 shows the curve of (*αE*_photon_)^2^ versus the photon energy *E*_photon_, where *α* and *E*_photon_ (hν) are respectively the absorption coefficient and the discrete photon energy. The *E*_g_ was determined by extrapolating the linear portion of the curve to zero, and the calculated value of *E*_g_ is 2.22 eV, which is a little larger than 2.17 eV for bulk materials. This may be attributed to the size effects since the microspheres are composed of nanoscaled particles, according to the SEM and TEM results.

**Figure 4:**
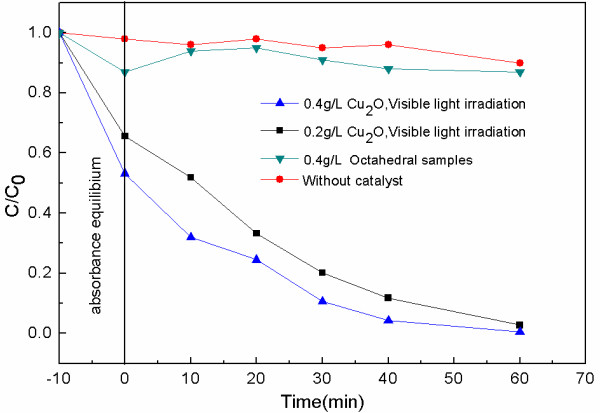
**Photocatalytic activity of the Cu**_**2**_**O microspheres under visible light irradiations in different concentrates.**

The photocatalytic activity of the Cu_2_O microspheres under visible light irradiations was also investigated. A cutoff filter was added under the Xe lamp to filter out the UV part (*λ* < 400 nm) to form a visible light source. The hollow porous Cu_2_O microspheres were dispersed in a MO aqueous solution and stirred for 10 min in the dark to establish absorption-desorption equilibrium. Then, the beaker containing this mixed solution was placed in the visible light source. An amount (3 ml) of the solution was removed from the beaker every 10 min to test the absorbance properties at 464 nm after filtrating the solid spheres. As shown in Figure 4, although the higher concentration initially degraded fast, two samples with different concentrations of 0.2 and 0.4 g/l were almost completely degraded in 1 h; especially for the sample with 0.2 g/l concentration, the degradation reached 80 % within 30 min. To confirm the results further, self-degradation experiment without hollow porous Cu_2_O microspheres was also performed, that is, only the MO solution was degraded under the same visible light source. Obviously, its degradation effect can be neglected. Compared with other reported results [35,36], hollow porous Cu_2_O microspheres possess higher degradation efficiency, that is, it has excellent visible light photocatalytic activity.

**Figure 5:**
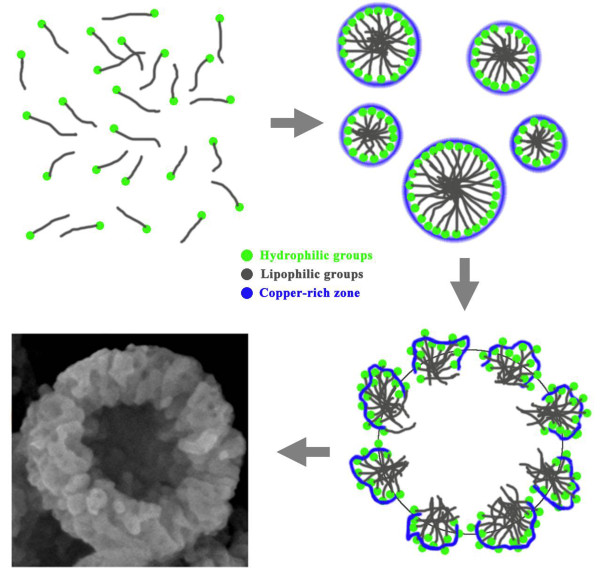
**SEM images. (a)** Products without SDS, **(b)** with ammonia amount increased from 0.04 to 0.08 ml, and **(c)** with NaOH amount increased from 0.15 to 0.3 ml.

This higher degradation efficiency of hollow porous Cu_2_O microspheres under visible light is mainly attributed to its peculiar structure with large specific areas as well as its outstanding visible light optical absorption characteristics. A hollow porous structure can provide more surface contact and space to absorb the pollutant molecule to make the excited electron arrive more easily at the surface [37]. As a comparison, the photocatalytic property of Cu_2_O particles with smooth octahedral surface (the SEM image is shown in Figure 5a) was also investigated. As shown in the result in Figure 4, its degradation efficiency is much less than those of the hollow porous samples, which indicates that the hollow porous structure played an important role for its higher degradation efficiency.

In order to confirm the above explanations, experiment without SDS was also performed. As shown in Figure 5a, some well-crystallized particles in octahedral shapes were formed instead of hollow porous microspheres, which indicate the soft-template role of SDS in the reaction.

The effects of ammonia and NaOH amount were also investigated. When the ammonia amount was increased from 0.04 to 0.08 ml (see Figure 5b) or the NaOH amount was increased from 0.15 to 0.3 ml (see Figure 5c), the morphology of the products changed a lot, and the hollow structure became imperfect. Especially, there formed many small nanoparticles and nanowires at the third condition (see Figure 5c). Summarily, SDS and the amount of reagents all played very important roles in the synthesis of novel hollow porous Cu_2_O microspheres.

**Figure 6:**
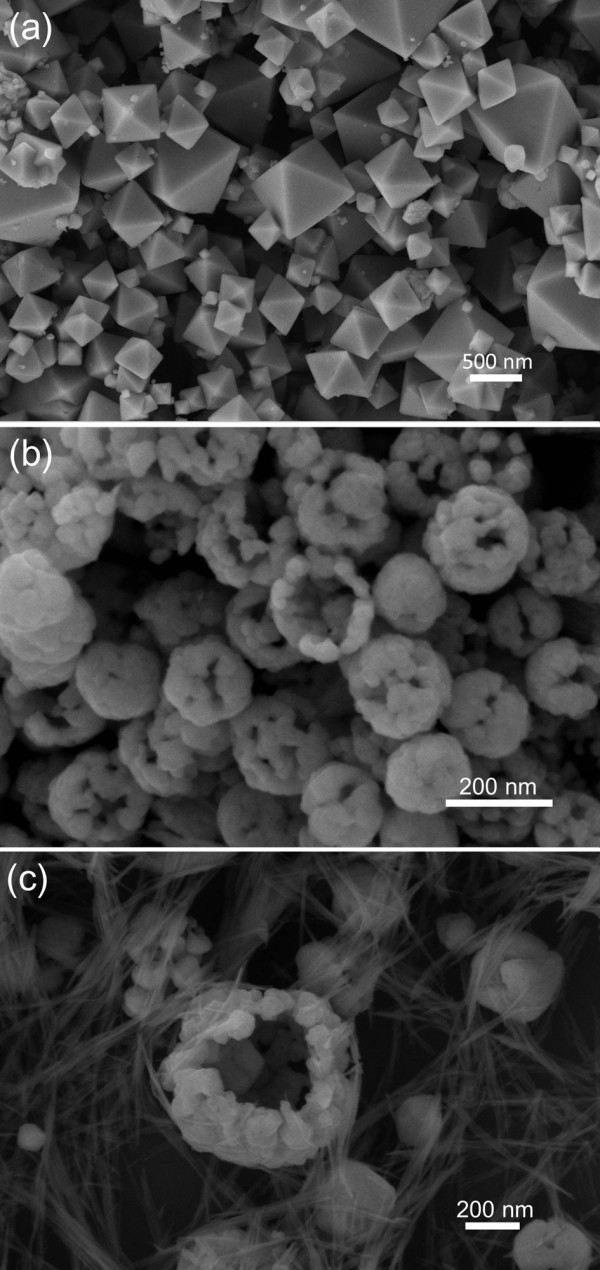
**Growth schematic of hollow porous Cu**_**2**_**O microspheres.**

The proposed growth process of hollow porous microspheres may be understood by the schematic shown in Figure 6. The anionic surfactant SDS acts the role of soft template and is a stable micelle in the solution. The inside lipophilic groups intertwined together in aqueous solution, whereas the outside hydrophilic groups containing sulfates attract Cu^2+^ to form the copper-rich zone. When ammonia and NaOH were added to the solution, Cu(OH)_2_ precursor formed firstly at the outside of the micelle and, finally, was reduced to Cu_2_O by hydrazine hydrate, which is a common reduction agent. At the same time, N_2_ was generated in the redox reaction process and produced lots of bubbles. Under the effects of SDS and bubbles, both of which acted as soft templates, the hollow porous Cu_2_O microspheres would be formed finally. The related chemical reactions should be as follows:

(1) $${\text{Cu}}^{2+}+{\text{ 2NH}}_{3}{\text{H}}_{2}\text{O}\to \text{Cu}{\left(\text{OH}\right)}_{2}+{2\text{NH}}^{4+}$$

(2) $${\text{Cu}}^{2+}+{\text{ 2OH}}^{-}\to \text{Cu}{\left(\text{OH}\right)}_{2}$$

(3) $$4\text{Cu}{\left(\text{OH}\right)}_{2}+{\text{N}}_{2}{\text{H}}_{4}\to {2\text{Cu}}_{2}\text{O}+{6\text{H}}_{2}\text{O}+{\text{N}}_{2}$$

## Conclusions

We have successfully synthesized hollow porous Cu_2_O microspheres with high purity by using a soft-template method at room temperature. This material has excellent photocatalytic activity under visible light irradiation in the degradation of MO owing to its unique optical properties and special morphology. The band gap was calculated to be 2.22 eV from its UV-visible absorbance spectrum. It was also found that the SDS acted as the soft template, and the amounts of ammonia and NaOH had important effects on the morphology of the products. These Cu_2_O microspheres with hollow porous structures may be a good candidate of photocatalytic materials under visible light irradiation.

## Competing interests

The authors declare that they have no competing interests.

## Authors' contributions

YY performed the experiment and measured the SEM and TEM data. LYZ designed the experiments and wrote the manuscript. ZY helped in the technical support for the experiments. JW and NTH participated in the measurements. MCL provided useful suggestions. YFZ supervised all of the study and provided financial support. All authors read and approved the final manuscript.
